# Juvenile osteochondritis dissecans in the lateral femoral condyle requiring osteochondral autograft as a revision procedure: a case report

**DOI:** 10.1186/s13256-015-0795-1

**Published:** 2016-01-14

**Authors:** Ryo Kanto, Hiroshi Nakayama, Tomoya Iseki, Shinichi Yoshiya

**Affiliations:** Department of Orthopaedic Surgery, Hyogo College of Medicine, 1-1 Mukogawa-cho, Nishinomiya, Hyogo 663-8501 Japan

**Keywords:** Bilateral, Knee, Osteochondritis dissecans

## Abstract

**Background:**

The optimal treatment option for osteochondritis dissecans of the knee is still controversial. We report the case of a boy who developed osteochondritis dissecans in the lateral femoral condyles of his bilateral knees requiring repeat surgical procedures. There has been no literature reporting juvenile osteochondritis dissecans of bilateral knees requiring repeat surgical procedures.

**Case presentation:**

A 6-year-old Japanese boy presented with pain in his bilateral knees. Although conservative treatment with prohibition of sports activities was continued for 6 months, healing could not be attained. Conservative treatment consisting of prohibition of sports activities that included running and jumping and use of a brace with a locking mechanism at full extension was applied. He was instructed to walk with the brace. Since his lateral femoral osteochondritis dissecans lesion was located at the contact area during flexion, weight bearing with the use of the brace could effectively unload the lesion. Surgery was subsequently conducted on his left knee which had a more advanced stage lesion. Transchondral drilling was performed because the articular surface maintained its smooth continuity. At 9 months after the surgery, no appreciable healing was observed in the follow-up radiographs. Moreover, during the postoperative time course, lesions suggestive of osteochondritis dissecans in his contralateral right knee had become more evident. Based on the diagnosis of delayed union of bilateral osteochondritis dissecans lesions, a second surgery was attempted. The preceding arthroscopic observation of his left knee showed preserved surface continuity with softening and suspected partial detachment. Considering the delayed healing process observed in this patient, autogenous cylindrical osteochondral graft transplantation (8 mm in diameter) was performed as a revision procedure, while transchondral drilling was performed for the stable osteochondritis dissecans lesion in his right knee. Postoperatively, healing was achieved at 6 months.

**Conclusions:**

Following failed conservative treatment, he underwent arthroscopic drilling; however, the osteochondritis dissecans lesion did not heal requiring revision surgery using a cylindrical autogenous osteochondral graft. Finally, clinical and radiological healing was attained 6 months after the second surgery. Initial presentation at a young age with bilateral lesions may be clinical factors related to poor healing response and susceptibility to stress-related subchondral lesions.

## Background

It is generally agreed that the prognosis of osteochondritis dissecans (OCD) of the knee in skeletally immature patients is favorable [1–4]. Therefore, the initial treatment option for a stable lesion is non-operative management including activity restriction, non-weight bearing, or use of brace or cast [1–6]. When the OCD lesion exhibits signs of poor healing or progression to unstable condition despite the non-operative treatment for 3 to 6 months, surgical intervention is considered.

Regarding the surgical option, drilling is generally indicated for stable lesions with the intention of promoting healing. Previous studies examining the results of drilling for stable lesions in skeletally immature patients showed a high healing rate ranging from 67 to 90 % [7–10]; however, postoperative-delayed healing requiring revision surgery was occasionally encountered [11]. In this situation, potential options for the second procedure vary depending on the status of the OCD lesion. Although internal fixation with bone graft is generally indicated as a primary procedure for unstable OCD lesions [1–4], the procedure options in revision surgery for the lesion with poor healing (or progression) following drilling have not been clarified due to its rarity.

We report the case of a young patient with an OCD lesion in his lateral femoral condyle who underwent cylindrical autogenous osteochondral graft following failed transchondral drilling. Subsequent to the second surgery, satisfactory clinical and radiological healing was attained at 6 months.

## Case presentation

A 6-year-old Japanese boy presented with pain in his bilateral knees. In addition to sports activities in his primary school, he had been practicing figure skating 5 days a week for 2 years. Although he did not complain that his knee pain caused a significant problem in his daily living activities, his sports activities were restricted by knee pain. He had no remarkable morbidities related to his knee in the past, and no family history relevant to musculoskeletal disorders.

A physical examination of his affected knee revealed full range of motion without swelling, instability or apparent malalignment. In addition, no abnormal findings indicating neurological and general musculoskeletal disorders were demonstrated. Laboratory data showed normal ranges in all examined parameters.

**Fig. 1:**
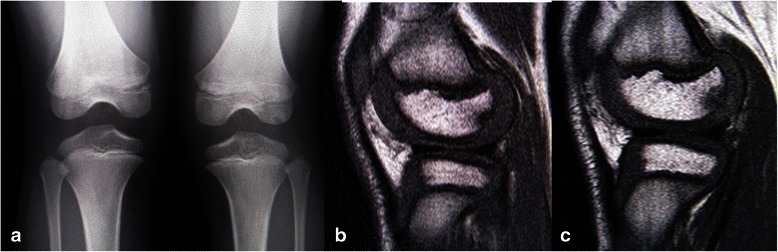
Image examinations at initial visit. **a** A posterior-anterior weight-bearing radiograph (Rosenberg’s view) at initial visit. An irregular radiolucent lesion with surrounding sclerosis is identified in the epiphyseal region. **b**, **c** T1-weighted sagittal magnetic resonance imaging images at first visit. An area of low signal intensity at the periphery of subchondral bone contour is present in the lateral femoral condyles of the patient’s right (**b**) and left (**c**) knees

A plain radiograph (posterior-anterior weight-bearing view) revealed a radiolucent lesion in the subchondral region coincident with the radiologic features of OCD in the lateral femoral condyle of his left knee (Brückl classification [1] Stage II), while no distinct radiological abnormalities were observed in his right knee. Subsequent magnetic resonance imaging (MRI) showed subchondral lesions suggestive of OCD in the lateral condyles of his bilateral knees, which demonstrated low signal lesion (defect) with reactive changes in the surrounding bone marrow. These lesions were classified as Stages I and II by Hefti’s classification [2] for the right and left knees respectively (Figs. 1a–c), and stage I by Bohndorf’s classification [12].

**Fig. 2:**
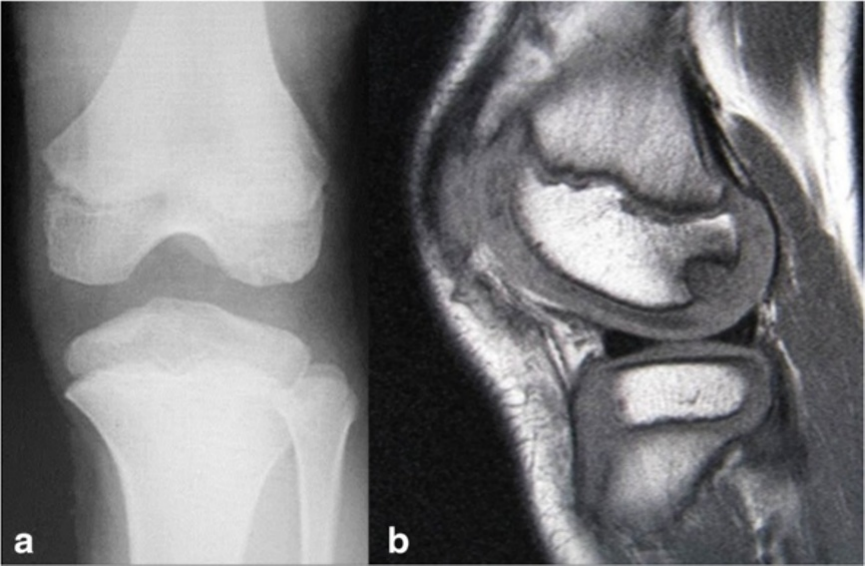
Image examinations at 6 months after the initial visit. **a** A posterior-anterior weight-bearing radiograph (Rosenberg’s view) of the patient’s left knee after 6 months of conservative treatment. **b** T1-weighted sagittal magnetic resonance imaging image. The osteochondritis dissecans lesion is evident

Conservative treatment with prohibition of sports activities was continued for 6 months. During this period, use of a hinged brace with locking mechanism was instructed while full weight bearing was allowed with the use of the brace. The brace was locked in extension while walking and the locking mechanism was released during sitting or non-weight bearing activities. Since the OCD lesion in the lateral femoral condyle is located at the contact area in flexion, use of the brace locked in extension could effectively unload the lesion during the weight bearing activities. We did not prescribe any physical treatment or medications. Although the conservative treatment was continued, satisfactory healing was not attained. The radiograph at 6 months after the initial visit showed no apparent healing (Fig. 2). Subsequently, surgical intervention was indicated for his left knee which had a lesion at a more advanced stage. At that time, he had no symptoms in his left knee due to the strict activity modification.

Preoperatively, length and direction of drilling were estimated on sagittal plane MRI images to avoid the risk for growth plate injuries. During surgery, the operative knee was deeply flexed to access the lesion located in the posterior area. Location of the drilling was intraoperatively monitored on lateral fluoroscopic images and confirmed by the postoperative MRI. The postoperative regimen was the same as applied in the conservative treatment.

At 9 months after the surgery, however, no appreciable healing was observed for the lesion in the follow-up radiograph of his left knee. Moreover, during the postoperative time course, the OCD lesions in the lateral and medial condyles of his contralateral right knee had become evident. Considering the prolonged treatment course with apparent poor healing observed for this patient, additional surgery was attempted for his bilateral knees at 11 months after the first surgery. Although there appeared to be possibilities of inherent weakness or constitutional factors predisposing him to epiphyseal lesions, neither radiological examination of other skeletal regions nor laboratory data indicated any associated abnormalities.

**Fig. 3:**
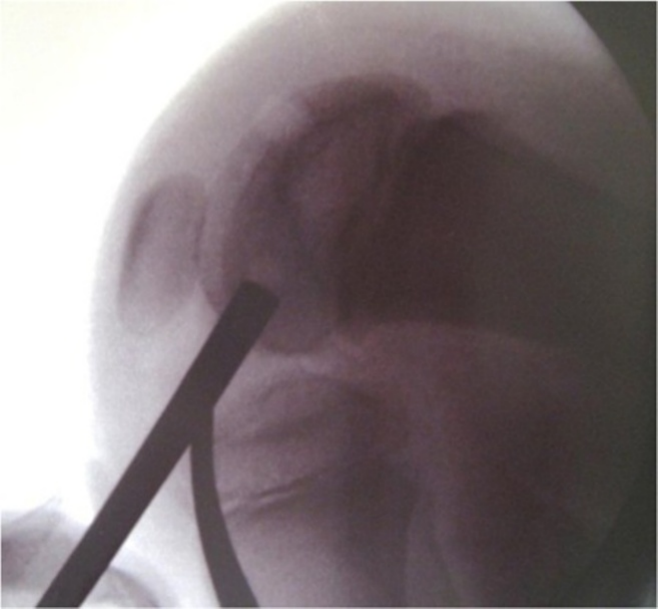
Fluoroscopic view during transplantation of the osteochondral graft

At the second surgery, arthroscopic observation of his left knee showed surface continuity with softening and suspected partial discontinuity on probing. Although the lesion did not reach the condition of detachment, autogenous cylindrical osteochondral graft transplantation (8 mm in diameter) was performed as a revision procedure considering the clinical course of this patient suggestive of inferior healing reaction. In the preoperative assessment, length and direction of the graft harvest and transplantation were determined on sagittal plane MRI images. At surgery, a 1.5-cm long osteochondral graft was harvested from the lateral femoral condyle just anterior to the terminal sulcus (contact area at full extension). During the harvest and transplantation procedures, we repeatedly checked the direction and distance from the growth plate on intraoperative fluoroscopic images (Fig. 3). The OCD lesions in his right knee were assessed to be grade I and transchondral drilling was performed on this side.

**Fig. 4:**
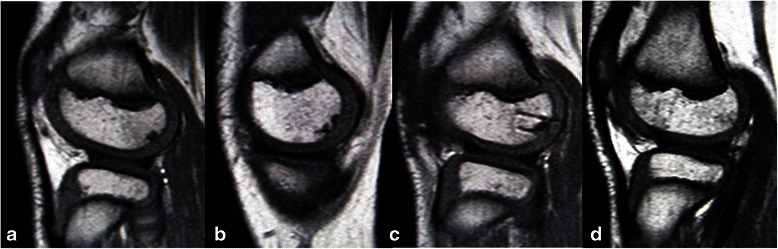
T1-weighted sagittal magnetic resonance imaging images at 1 year and 3 months after the second surgery. **a** Lateral femoral condyle of the patient’s right knee following transchondral drilling. **b** Medial femoral condyle of his right knee following transchondral drilling. **c** Lateral femoral condyle of his left knee following cylindrical osteochondral grafting. **d** Graft donor site in the lateral femoral condyle

**Fig. 5:**
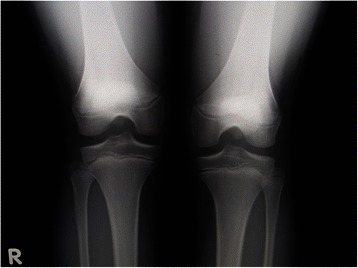
A posterior-anterior weight bearing radiograph (Rosenberg’s view) of the patient’s left knee at 3 years and 6 months after reoperation showing radiological healing of the osteochondritis dissecans lesions in both knees

Postoperatively, the same management as for the first surgery was applied. The grafted lesion in his left knee showed radiological signs of healing in 6 months while satisfactory healing of the OCD lesions on his right side was also achieved by 6 months. Subsequently, sports activities were gradually resumed; he did not experience any physical limitations with full performance of this knee. MRI at 1 year and 3 months after reoperation showed findings indicative of healing in his bilateral knees (Figs. 4a–c). During the subsequent period, he continued to be asymptomatic. The final follow-up evaluations at 3 years and 6 months after the second surgery showed no abnormal findings in a physical examination with complete healing of the subchondral bone lesion in the radiograph (Fig. 5).

## Discussion

OCD of the knee is thought to be a subchondral lesion caused by excessive load applied to the knee during activities. Based on the age at onset, the lesion is classified into juvenile and adult forms. The age at initial presentation predominantly ranges from 10 to 15 years, and the male-to-female ratio has been reported to be approximately 2:1. With the recent trend of increasing participation in strenuous sport activities at younger ages, the mean age of onset has been decreasing [2–5]. Although the “classic” site of the medial femoral condyle is most frequently affected accounting for more than 70 % of cases, lateral femoral condyle lesions are equally encountered in our patient population due to increased prevalence of lateral discoid meniscus in our country [6]. The present case represents the youngest case among the previously reported patient population. Strenuous figure stating practice starting at the age of 4 years may have given rise to excessive stress to his cartilage and underlying immature bony tissue in this case.

Regarding the treatment, it has been reported that the results of conservative treatment in juvenile OCD with open epiphysis are generally favorable. The reported rate of healing ranges from 50 to 81 % [1, 5, 6]. Although a variety of conservative measures, including cast immobilization, use of crutches and non-weight bearing activity modification have been reported, Hefti *et al*. reported that no difference was found among the different treatment methods based on the results of a European multicenter study [2]. Reported clinical factors associated with poor prognosis are lesion size (more than 20 mm in diameter) and symptoms of pain and/or swelling at the initial presentation [3, 5]. Reports of the effect of location (medial or lateral condyle) on healing rate are contradictory among the literature. A multicenter study by Hefti *et al*. indicated an inferior healing rate in lateral condyle lesions [2], whereas all lateral lesions were healed by conservative treatment in contrast to 61 % healing rate for medial lesions in the case series by Wall *et al*. [7]. Our patient had knee pain at initial presentation and the lesion size was approximately 1 cm in diameter located in the lateral femoral condyle. Although the clinical characteristics may not particularly indicate poor prognosis, specific factors observed in this case such as onset at young age and bilateral occurrences seem to imply involvement of inherent factors affecting the healing process.

The surgical options for cases with failed conservative treatment include drilling as an initial option for stable lesions. The reported healing rate following drilling is generally high ranging from 67 to 90 % [7–10]; however, failure of healing after drilling procedure has been occasionally encountered. In this situation, possible options for revision surgical procedures include repeat drilling, bone grafting, bone peg fixation, and internal fixation with bone graft. If no healing is achieved after drilling as in this case, some forms of bone grafting should be considered for revision. Use of an autogenous osteochondral graft has been reported for treatment of unstable OCD lesions, after which both biological healing enhancement and mechanical stabilization are expected [11, 13–17]. Miniaci and Tytherleigh-Strong reported the successful results of cylindrical autogenous osteochondral grafting for three patients for who an initial attempt at arthroscopic drilling failed [14]. This procedure could afford a satisfactory healing response in the present case which has an unusual clinical presentation. However, we have to pay attention to the potential risk for complications associated with an osteochondral autograft procedure, such as donor cite pain, a patellofemoral problem, and possibility of growth disturbance caused by growth plate injury. Regarding the image assessment during the follow-up period, a computed tomography (CT) examination in addition to serial radiographs would have provided more accurate information.

## Conclusions

We report the case of a 6-year-old boy who developed OCD of the lateral femoral condyles in bilateral knees. Following failed conservative treatment, he underwent arthroscopic drilling; however, the OCD lesion did not heal requiring revision surgery using a cylindrical autogenous osteochondral graft. Finally, clinical and radiological healing was attained 6 months after the second surgery. Initial presentation at a young age with bilateral lesions may be clinical factors related to poor healing response and susceptibility to stress-related subchondral lesions.

## Consent

Written informed consent was obtained from the patient’s legal guardian(s) for publication of this case report and any accompanying images. A copy of the written consent is available for review by the Editor-in-Chief of this journal.
